# Live bird markets as evolutionary epicentres of H9N2 low pathogenicity avian influenza viruses in Korea

**DOI:** 10.1080/22221751.2020.1738903

**Published:** 2020-03-17

**Authors:** Sung-su Youk, Dong-Hun Lee, Jei-Hyun Jeong, Mary J. Pantin-Jackwood, Chang-seon Song, David E. Swayne

**Affiliations:** a Exotic and Emerging Avian Viral Diseases Research Unit, Southeast Poultry Research Laboratory, U.S. National Poultry Research Center, Agricultural Research Service, U.S. Department of Agriculture, Athens, GA, USA; b Avian Diseases Laboratory, College of Veterinary Medicine, Konkuk University, Seoul, Korea; c Department of Pathobiology & Veterinary Science, University of Connecticut, Storrs, CT, USA

**Keywords:** Avian influenza, Korean H9N2, live bird market, evolution, reassortment, ecology

## Abstract

Live bird markets (LBMs) in Korea have been recognized as a reservoir, amplifier, and source of avian influenza viruses (AIVs); however, little was known about the role of LBMs in the epidemiology of AIVs in Korea until recently. Through 10 years of surveillance (2006–2016) we have isolated and sequenced H9N2 viruses in Korean LBMs. To understand how H9N2 evolves and spreads in Korea, a statistical Bayesian phylogenetic model was used. Phylogenetic analysis suggests that three separate introductions of progenitor gene pools, Korean domestic duck-origin and two wild aquatic bird-origin AIVs, contributed to the generation of the five genotypes of H9N2 viruses in Korea. Phylogenetic reconstruction of ecological states infer that the LBMs are where chickens become infected with the virus, with domestic ducks playing a major role in the transmission and evolution of the H9N2 viruses. Three increases in the genetic diversity of H9N2 viruses were observed and coincided with transitions in host species and the locations (domestic farm, LBM, slaughterhouse, and wild aquatic bird habitat) where the viruses were isolated, accompanying genetic reassortment. Following the introduction of a wild aquatic bird-origin AIVs in 2008, six genes of the Korean lineage H9N2 virus were replaced with genes originating from wild aquatic birds, and viruses with this new genotype became predominant in Korean LBMs.

## Introduction

Wild aquatic birds are the natural reservoir of avian influenza viruses (AIVs), as they can have a variety of combinations of hemagglutinin (HA) and neuraminidase (NA) virus subtypes and show no or minimal clinical signs of infection [1]. H9N2 has become the most prevalent subtype of low pathogenic avian influenza in poultry. After introduction to Eurasian poultry, this virus has diverged into five genetic lineages: G1, Y280, BJ94, G9, and Y439/Korea lineage [2] and has become endemic in poultry across Asia, the Middle East, and North Africa [3].

The segmented nature of the AIV genome permitted the reassortment of H9N2 viruses with other concurrently circulating AIVs that contributed to the emergence of H5N1 highly pathogenic avian influenza (HPAI) [4,5], as well as zoonotic H7N9 and H10N8 viruses in China [6–8]. In contrast, there have been instances that H9N2 viruses received single or multiple genes from H5N1 and H7N3 HPAI viruses in Bangladesh, Pakistan, and China [9–11], and some of these H9N2 viruses showed increased zoonotic potential as well as adaptation to poultry species [12,13].

The first identification of the H9N2 virus in South Korea confirmed in a commercial chicken farm in 1996 [14]. Phylogenetic analysis revealed that the HA gene of the H9N2 viruses were closely related to A/duck/Hong Kong/Y439/97 (Y439) lineage viruses [15]. Since then, the virus became endemic in domestic poultry in Korea and has formed a distinct Korean lineage [2]. The H9N2 viruses have evolved through reassortment with AIV from wild aquatic birds and domestic ducks, generating new genotypes [16–19]. The Korean government authorized the use of an inactivated H9N2 vaccine since 2007, and vaccination has been implemented in commercial chickens. Although vaccination was successful in reducing disease prevalence and economic loss, the Korean H9N2 viruses further diverged into a distinct genetic group away from the vaccine strain [20].

A comprehensive understanding of the evolution and ecological dynamics of H9N2 viruses remains unclear due to limited genetic information. Here, we isolated H9N2 viruses from LBMs and conducted whole genome sequencing which enabled us to perform refined phylogenetic analyses with the addition of pre-existing genetic information from the public database. Furthermore, we integrated ecological and genetic data into Bayesian phylogenetic models to infer the dispersal and genetic diversity of H9N2 viruses in Korea.

## Material and methods

### Sample preparation and sequencing

Between 2006 and 2016, 4806 samples were collected from LBMs in South Korea. Oropharyngeal and cloacal swabs and tissue homogenates (trachea and cecal tonsil) were suspended in phosphate-buffered saline containing 1% gentamicin and used for virus detection. AIV identification was conducted by chicken embryonated egg inoculation and real-time reverse transcription PCR targeting the matrix gene using a standard diagnostic protocol [18]. Presence of AIV was confirmed by further nucleotide sequencing of HA and NA genes from the matrix gene-positive samples. Allantoic fluids confirmed with AIV were stored at −80°C until sequencing. Among the 127 AIV-positive samples confirmed by identification of the subtypes, the H9N2 subtype were the most prevalent (*n* = 94; 74.0%), followed by H3N2 (*n* = 16; 12.6%) and H3N8 (*n* = 5; 3.9%). We selected 104 samples best representing the periodical and geographical diversity of the AIV-positive samples confirmed, the detailed data of which were available in the supplemental file. Samples were subjected to viral RNA extraction using MagNA Pure 96 DNA and Viral NA kits (Roche Diagnostics). All eight segments of cDNA were synthesized and amplified via reverse transcription PCR using the OneTaq® One-Step RT–PCR Kit (New England Biolabs) [21]. The Nextera XT DNA Sample Preparation Kit (Illumina, USA) was used to generate multiplexed paired-end sequencing libraries according to the manufacturer's instructions. Barcoded multiplexed library sequencing was performed on an Illumina MiSeq platform (Illumina). The sequences of viruses isolated were reconstituted by *de novo* and directed assembly using the Geneious 10.0.9 software and GALAXY platform [22]. The GALAXY platform accommodated the MIRA3 algorithm that generate a consensus sequence by using de-novo assembly. We set up the highest standard (switch for assemble quality grade: accurate) in assembling sequences, which picks up nucleotides polymorphism below 99% consensus and transcribe a set of separate contigs. For those H9N2 viruses having more than one consensus sequence, we manually repeated assemblies by mapping the original NGS reads to the resulting contigs using the Geneious software. A consensus sequence with a higher mean coverage (*p* < 0.01) was reported as a definitive sequence and used for following phylogenetic analysis. We found only one sample of mixed infection with H9N2 and H4N6 virus. We reported this isolate as an H4N6 subtype, A/White Pekin duck/Korea/N08-0187/2008(H4N6), because the assembled data showed that the mean coverage of the H9N2 virus (Mean ± Std Dev = 50.8 ± 25.3 for H9 HA; Mean ± Std Dev = 29.2 ± 16.9 for N2 NA, total number of the reads for H9 and N2 was 373 (0.15% of total reads) and 197 (0.08% of total reads)) was significantly lower than H4N6 virus (Mean ± Std Dev = 399.6 ± 158.3 for H4 HA; Mean ± Std Dev = 3121.8 ± 1537.2 for N6 NA, total number of the reads for H4 and N6 was 2954 (1.2%) and 19823(8.2%) of the total 241,278 reads). We did not use the genome sequences of this virus in the H9 and N2 phylogenetic analysis. Consensus sequences have been deposited in GenBank with collection date, host species, collection sites (Supplemental file).

### Data collection and phylogenetic analysis

The sequences of all eight gene segments of AIVs isolated in Korea from 1996 to 2016 were retrieved from the Influenza Research Database (www.FluDB.org). H9N2 viral sequences related to the Korean isolates were searched by using the BLAST function (Geographic grouping: China, Japan, Mongolia, and Vietnam; Access date: August 2018). To identify the genetic correlation of Korean H9N2 AIV with viruses from Eurasia and North America, global phylogenetic trees were created by the Maximum-likelihood (ML) algorithm using sequences from the BLAST results and sequences pruned using the software CD-HIT with a 90% sequence identity level to filter-out representative sequences of AIV identified from 1970 to 2016 [23]. Sequences with < 90% of the full length were removed. Sequence alignments for each segment were generated using MUSCLE v3.8.31 [24]. We manually examined alignments to ensure accuracy and retained only the coding region for phylogenetic analyses. In total, 4710 sequences were processed for phylogenetic analysis, including AIV sequences identified in this study (*n* = 832), published Korean AIV sequences (*n* = 3201), and sequences genetically related to the Korean H9N2 by the BLAST and the representative sequences for Eurasia and North America (*n* = 645). The sequences were used to construct ML trees for each gene using RAxML ver 8.2.12 [25]. Bootstrap support values were generated using 1000 rapid bootstrap replicates. TempEst v1.5.1 was used to identify potential outliers that substantially deviated from the linear regression of root-to-tip genetic distance against time, and the outliers were removed from this study [26]. Genotypes were designated with bootstrap values over 70% and genetic clustering as follows: Korean lineage (KOR), Y439 lineage (Y439), and Eurasian gene pool lineage (EGP).

### Genotype classification of H9N2 viruses

Genotypes of H9N2 viruses identified in Korea from 1996 to 2016 were analysed. Viruses were genotyped if sequences and genetic lineage assignments were available for all eight segments. To identify progenitors of viral segments of H9N2 viruses, a possible progenitor gene pool was assigned according to the following criteria: (1) the progenitor gene pool and H9N2 viruses are located in the same genetic cluster with a minimum bootstrap value of 70; (2) a descendant of the progenitor gene pool is a monophylogeny and not a year apart from the first introduction of H9N2 virus; and (3) the progenitor gene pool includes a minimum of one isolate from the Korean wild aquatic birds or domestic avian species. If not, based on the genetic distance of HA, a genotype of the most closely related isolate was designated as a surrogate.

### Time-scaled Bayesian phylogenetic analysis

Statistical selection of models of nucleotide substitution was carried out using jModelTest for the alignment of all eight segments [27]. The Akaike Information Criterion and Bayesian Information Criterion were used to perform the model selection (Supplemental table 1). To estimate the time to the most recent common ancestor (TMRCA) and demographic history of each segment, we inferred time-scaled phylogenies by Bayesian Markov Chain Monte Carlo (MCMC) sampling using BEAST v1.10.1 [28]. Sequences with isolated dates (exact month, day, and year) were used for each gene segment. For sequences that are available only with the year of isolation, an isolated date was estimated by sampling dates within the years. We used uncorrelated log-normal relaxed clock models and Gaussian Markov random field (GMRF) Bayesian skyline coalescent tree prior in the analyses [29]. Three independent runs were combined for analyses to ensure that an adequate effective sample size (200) was reached for relevant parameters. The maximum clade credibility trees were estimated from a posterior (*posterior* > 0.5) distribution of trees with TreeAnnotator v1.10.1 and visualized using FigTree v1.4.4

### Estimation of viral exchange history between ecosystems

A discrete-trait phylodynamic model was adopted to estimate the history of viral transmission in Korea. All Korean H9N2 genes and sequences closely related by BLAST search were used for analysis (Supplemental file 1). The location of detection (commercial domestic farm, LBM, slaughterhouse, and wild aquatic bird habitat) and host type (chicken, duck, wild aquatic bird, and other avian species) were defined in the model as discrete nominal categories, including domestic-chicken, domestic-duck, LBM-chicken, LBM-duck, LBM-others, slaughter-chicken, slaughter-duck, and wild aquatic birds [30]. We used an asymmetric substitution model with the Bayesian stochastic search variable selection and a strict clock model. The same parameters were used to estimate changes in genotypes. The five genotype classifications were defined in the model as discrete nominal categories. At least 3 independent Markov chains were performed to meet the basis of the criteria of an effective sampling size of >200 as calculated by Tracer version 1.5 with a 10% burn-in (http://tree.bio.ed.ac.uk/software/tracer/). A maximum clade credibility tree was generated for each data set by using TreeAnnotator (http://www.phylo.org/index.php/tools/treeannotator.html). FigTree 1.4.4 (http://tree.bio.ed.ac.uk/) was used for visualization of trees. The mean transition rate and corresponding indicator were calculated from the resulting log files. Bayes factor (BF) support was calculated with the indicator to assess statistical support. Significant transitions between host groups were determined based on the combination of a BF > 3 and a mean indicator > 0.5. Support levels were defined as support (3 < BF < 10), strong support (10 < BF < 100), very strong support (100 < BF <1000), and decisive support (BF > 1000). To better characterize the expected ratio of traits and genotypes, we also employed posterior inference of the complete Markov jump and reward history of the HA gene through time [31]. The duration that a particular ecological or genotypic state was observed before transitioning to another state was recorded on a branch-by-branch basis from the posterior sampling of phylogenetic trees. To correlate the reconstituted ecological state and genotype, posterior probabilities (*k* > 0.5) were also extracted from internal nodes in order to correlate them.

## Results

### Genotypes of H9N2 viruses in Korea

**Figure 1.**
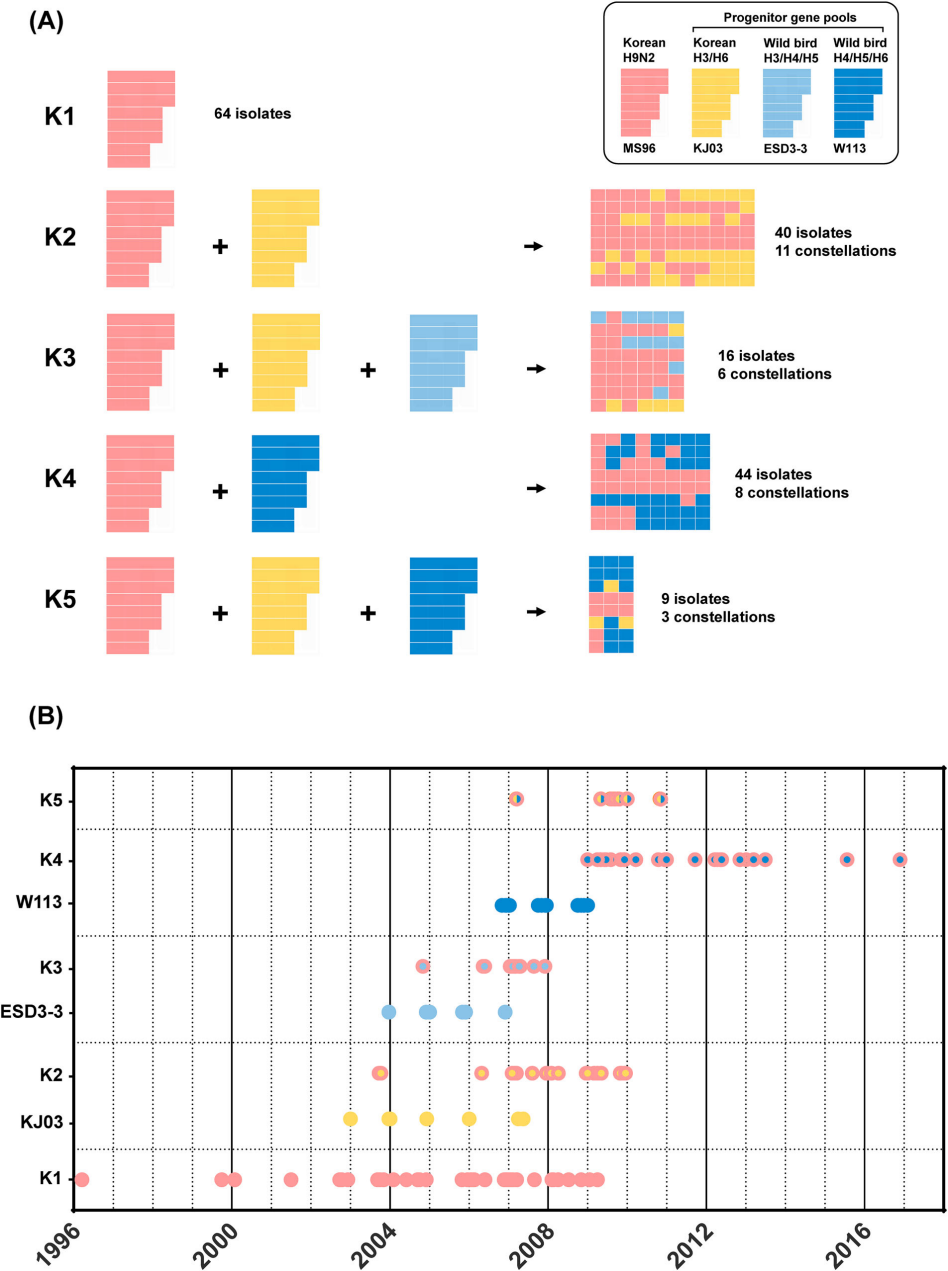
Genotype classification and time of virus isolation. Korean H9N2 virus (MS96) were designated as the backbone, and three progenitor gene pools (KJ03, ESD3-3, and W113) were assigned. (A) Each reassortment event generates different genome combinations. (B) The time of isolation of the three progenitor gene pools were followed by the generation of each genotype.

To examine the genetic diversity and evolution of the H9N2 viruses, the 864 gene segments sequenced in this study from 104 AIV isolates were added to the alignment of pre-existing Korean and related wild aquatic bird-origin AIV sequences. Based on the global ML phylogeny of each gene (Supplemental figure 1), twenty-nine genome constellations were identified. Genome constellations correlating with the ML phylogenetic tree of the HA gene show that only the HA and NP genes of Korean lineage were consistent except the A/silky chicken/Korea/N07-0400/2007(H9N2) strain, which had the NP gene originating from wild aquatic birds (Supplemental figure 3). Also, there was one heterogeneous NA reassortment with the N8 subtype (A/chicken/Korea/04164/2004) other than N2. We categorized these genome constellations into 5 genotypes based on the sources of three progenitor gene pools (KJ03, ESD3-3, and W113) (Figure 1). The initial Korean H9N2 viruses, represented by A/chicken/Korea/MS96/96 (MS96 group), was designated as an independent cluster (Korean lineage).

The H9N2 viruses composed of eight segments of Korean lineage were designated as genotype K1. Sixty-four K1 isolates were isolated in domestic farms (*n* = 35), LBMs (*n* = 28), and slaughterhouse (*n* = 1) between 1996 and 2008. Genotype K2 was produced by reassortment between MS96 and KJ03 groups, consisting of 40 isolates with 11 patterns of genome constellations. Since the first identification of K2 [16], it became the most prevalent in LBMs (*n* = 32) until 2009. Genotype K3 was produced by reassortment of the MS96, KJ03, and ESD3-3 groups which was first identified in 2004 as of a H9N8 subtype consisting of six genes (PB2, PA, NP, NA, M, and NS) from the ESD3-3 group and two genes (PB1 and HA) from the MS96 group [17,32]. Sixteen isolates of genotype K3 were identified with 6 genome constellations between 2004 and 2007. After consistent introduction of the ESD3-3 group through wild aquatic birds between 2004 and 2007, 17 isolates recurred between 2006 and 2007 with further reassortment with the PB1 and NS genes of KJ03 group. In particular, 12 out of 17 isolates were involved in the reassortment with the ESD3-3 group, which had PB2 and PA genes closely related to the A/wild/duck/Taiwan/WB551/2005 strain (Supplemental figure 2). Genotype K4 (K4) emerged from reassortment between the MS96 and W113 groups. After frequent detections of the W113 group in Korean wild aquatic birds between 2006 and 2008, 44 isolates with 8 genome constellations were identified between 2009 and 2016. Among them, 31 isolates were in LBMs, which makes it the second most prevalent genotype in LBMs. Genotype K5 (K5) was generated by reassortment of MS96, KJ03, and W113 groups. Nine isolates with 3 genome constellations were identified between 2009 and 2010. Collectively, three progenitor gene pools contributed to the emergence of novel genotypes of H9N2 viruses in LBMs where the newly emerged genotypes (K2–K5) were more widespread than the Korean lineage (K1); 78 of 106 LBM isolates were in K2–K5.

#### (1) Reassortment of H9N2 viruses with H3 and H6 viruses originating from Korean domestic ducks

The KJ03 progenitor gene pool consists of H3 and H6 viruses isolated from Korean domestic ducks. The first isolation, represented by A/duck/Korea/KJ/2003(H3N2), was in domestic ducks in 2003. The PB2, PB1, PA, NA, M, and NS genes of the KJ03 group contributed to the emergence of K2 and K5 (Figure 1). The PB2, PB1, M, and NS genes of KJ03 originated from early Korean H9N2 viruses (MS96 group). Then, they branched out and underwent reassortment with H3 and H6 viruses in domestic and LBM ducks (Supplemental figure 1). The mean TMRCA estimates for each gene segment of the KJ03 group indicated that the gene segments detected in Korean H9N2 viruses were likely to have emerged between August 1999 and November 2003. After circulation with H3 and H6 viruses, the four genes were reintroduced to the H9N2 viruses in 2006. The PA and NA genes of the Y439 lineage were introduced to KJ03 and subsequently to Korean H9N2 viruses. Due to the limited wild aquatic bird isolates in Korea in the 1990s, no related ancestor virus to KJ03 was available in the Korean wild aquatic birds. The mean TMRCA of the Y439 lineage segments of KJ03 in Korean poultry was estimated to be November 2002 for PA and January 1996 for NA, suggesting that the PA and NA genes of H3 and H6 viruses were present in domestic and LBM ducks prior to the reassortment with H9N2 viruses.

#### (2) Reassortment of H9N2 viruses with H3, H4, and H5 viruses originating from wild aquatic birds

The ESD3-3 progenitor gene pool for the H9N2 viruses was composed of viruses of H3, H4, and H5 subtypes originating from wild aquatic birds. These viruses had been detected periodically in Korean wild aquatic birds in winter seasons between 2004 and 2007. The first isolate was the A/aquatic bird/Korea/ESD3-3/2004 (H3N8) strain, representing ESD3-3 group. The PB2, PA, NP, and M genes of the ESD3-3 group contributed to the emergence of genotype K3 (Figure 1). These four genes clustered with the Eurasian wild aquatic bird gene pool (EGP) lineage, which was primarily identified in wild aquatic birds and domestic ducks in China and Taiwan as well as in domestic ducks in Korea (Supplemental figure 1). There were two H9N2 viruses having the NP or M gene of ESD3-3. The mean TMRCA of ESD3-3 in Korean wild aquatic bird was estimated to be November 2002 for PB2 and October 2002 for PA. The phylogenetic analysis suggest that the ESD3-3 progenitor gene pool was widely dispersed in AIVs originating from wild aquatic birds in East Asia before its introduction to Korean H9N2 viruses.

#### (3) Reassortment of H9N2 viruses with H5, H6, and H7 viruses originating from wild aquatic birds

The first identification of the W113 group genes in Korean poultry was A/duck/Korea/A14/2008 (H5N2) in domestic ducks. The W113 progenitor gene pool was composed of H5, H6, and H7 subtypes originating from wild aquatic birds. These viruses have been detected sporadically in Korean wild aquatic birds in the winter season between 2006 and 2008. The first isolation of this gene pool was the A/aquatic bird/Korea/W113/2006 (H5N2) strain, representing the W113 group. A phylogenetic analysis suggests that the PB2, PB1, PA, NA, M, and NS genes of the W113 group contributed to the emergence of K4 and K5 (Figure 1). The PB2 and M genes of the W113 group originated from the Y439 lineage. The mean TMRCA for W113 was estimated to be January 2002 for PB2 and January 2000 for M (Supplemental table 2). The PB1, PA, NA, and NS genes clustered with the EGP lineage and as primarily isolated from wild aquatic birds in Korea as well as from wild aquatic birds and domestic ducks in China (Supplemental figure 1). The mean TMRCA of the PB1, PA, NA, and NS genes in Korean wild aquatic birds was estimated between February 2001 and September 2004.

### Genetic diversity and ecosystem interactions

**Figure 2.**
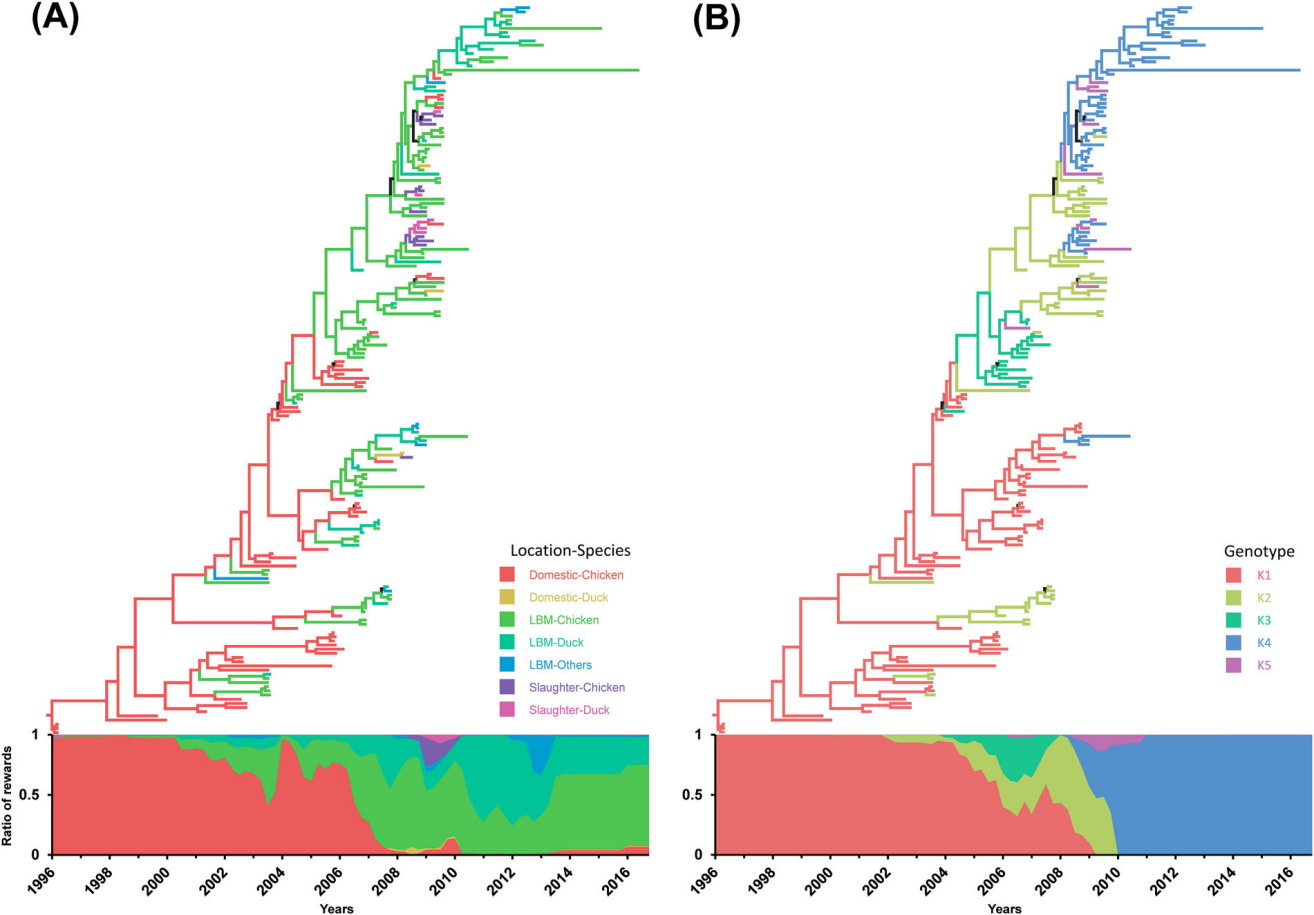
Estimation of changes in ecological states and genotypes on a time-scaled phylogenetic tree. Ecological states (A) and genotypes (B) of H9N2 viruses were reconstituted on a discrete state space and visualized on a time-scaled phylogenetic tree of the HA gene. The graph attached to the trees shows the proportional Markov jump reward of ecological state (A) and genotypes (B) over time.

To estimate changes in ecological states and genotypes of H9N2 viruses, HA genes were used for Bayesian phylogenetic framework (Figure 2). The domestic-chicken state was identified as the dominant trunk between 1996 and 2006, except the transient high portion of the LBM-chicken state in 2003. Consequently, LBM-chicken and LBM-duck states likely act as the dominant source of viruses. There was also a short-lived identification to slaughter-chicken and slaughter-duck states in 2009. For the genotypes, genotype K1 consisting of all Korean lineage segments was dominant between 1996 and 2003. After the introduction of KJ03 and ESD3-3 genes to the H9N2 virus, genotype K2 and K3 became dominant between 2004 and 2007. Genotype K4 became the only genotype detected in Korea after 2011. Genotype K5 was detected transiently between 2006 and 2011 at a low level.

**Figure 3.**
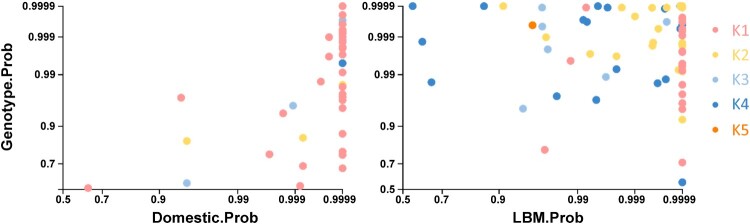
Posterior probabilities of ecological states and genotypes extracted from internal branches of the HA Bayesian phylogenetic tree. Posterior probabilities in the same Bayesian phylogenetic tree matching ecological state and genotype were extracted and plotted.

To further corroborate where the reassortment events occurred to produce the novel genotypes, posterior probabilities of ecological (domestic and LBM) and genotype states were extracted from internal branches of the Bayesian phylogenetic tree and plotted (Figure 3). The correlation of posterior probability supports that the generation of the new genotypes primarily took place in LBMs. In particular, three genotypes existed in the domestic state, including mostly genotype K1 (*n* = 44) and a few K2 (*n* = 3) and K3 (*n* = 6) genotypes. In contrast, the number of K2–K5 genotypes was higher in the LBM state (*n* = 75) compared to the number of K1 genotypes (*n* = 19). In conclusion, predominance of H9N2 in chickens in domestic farms shifted to chickens and ducks in LBMs, and then the reassortment of H9N2 viruses (genotype K2–K5) took over the initial Korean lineage H9N2 (genotype K1) viruses.

### Live bird markets as a potential reservoir of AIV

**Figure 4.**
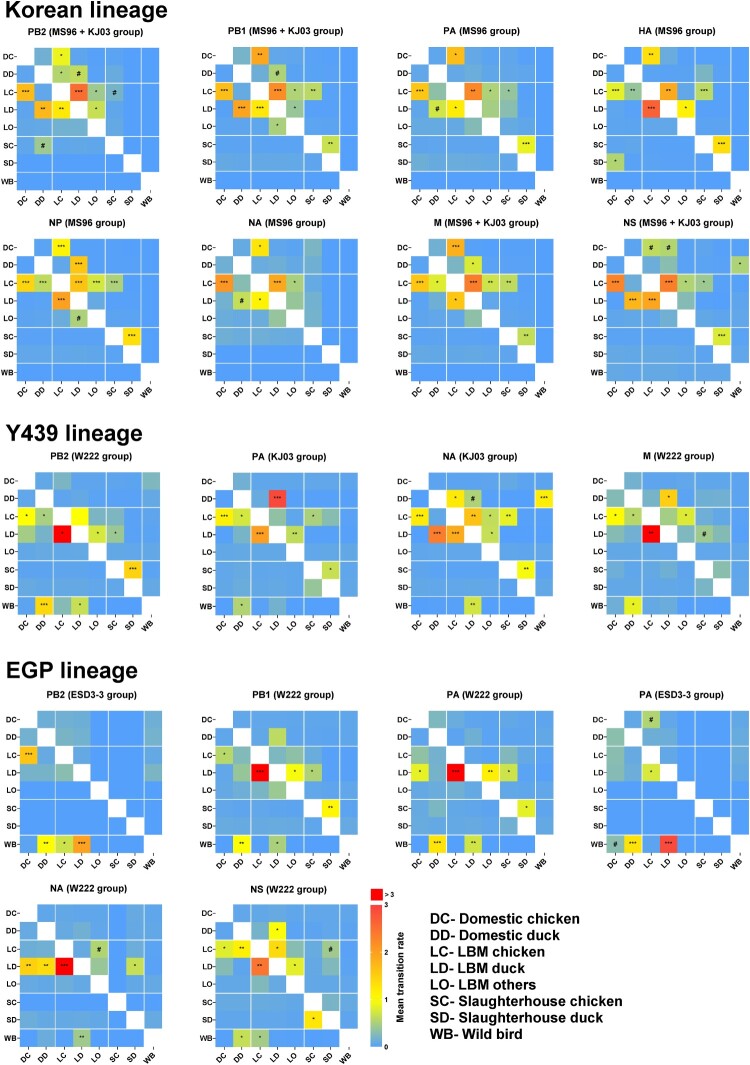
Heat maps showing source–sink dynamics and mean transition rates between ecological states (0–3.0+). The Y- and X-axes represent the source and sink, respectively. All statistically supported interactions are marked with special symbols (#, 3 < BF < 10; *, 10 < BF < 100; **, 100 < BF < 1000; ***, BF > 1000).

The transitions between ecological states were examined for all eight genes by inferring changes of discrete traits on the Bayesian phylogenetic tree (Figure 4). In the Korean lineage, well-supported transitions between domestic chickens and LBM chickens in all genes consistently provide support for the exchange of viruses between LBM and domestic chicken farms. The LBM-chickens state was well-supported as the source of infection of other ecological states (Domestic chicken, Domestic duck, LBM duck, LBM others, and Slaughterhouse chicken) after establishment of the virus in LBM chickens. In the Y439 lineage, transmission from wild aquatic birds to domestic- or LBM-duck was supported in PB2, PA, NA, and M genes that had reassortment with the H9N2 virus. In contrast to the Korean lineage, transmission from LBM-duck to LBM-chicken was more frequent than the opposite direction. In the EGP lineage, the transitions from wild aquatic bird to domestic/LBM ducks suggest that the domestic ducks were likely involved in the introduction and transmission of genes originating from wild aquatic bird to land-based poultry. The transition rates from LBM-duck to LBM-chicken in PB1, PA (EGP1 and EGP2), NA, and NS genes were higher than those in the opposite direction. In conclusion, viruses originating from wild aquatic birds (Y439 and EGP lineages) were adopted and redistributed by ducks in domestic farms and LBM, and Korean H9N2 viruses (Korean lineage), that spread to LBMs through domestic chickens, concurrently circulated in LBMs, which provided opportunities for the shuffling of their gene segments.

### Increase in the genetic diversity of H9N2 viruses with ecological changes and introduction of viruses originating from wild aquatic birds

**Figure 5.**
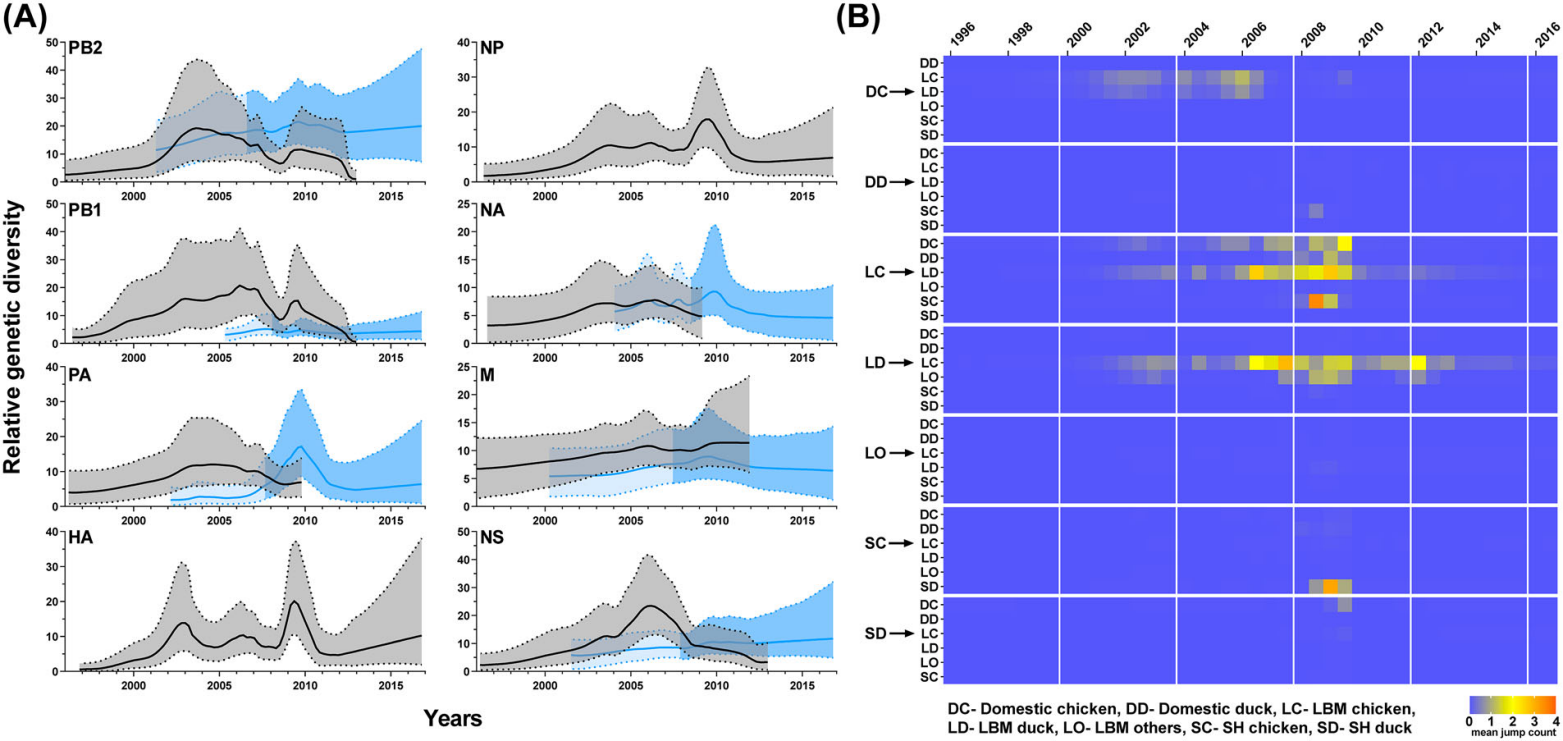
Gaussian Markov random field Bayesian skyline plot of eight genes from H9N2 viruses and time-delimited Markov jump count of the HA gene. (A) Each segment of the H9N2 virus was used to calculate the relative genetic diversity over time. Linear lines and shaded areas bounded by dotted lines represent the mean relative genetic diversity and 95% highest posterior density value, respectively. Black lines and adjacent grey areas represent the MS96 group. Blue lines and the adjacent blue area represent the W113 group. The blue shaded area with contrast separate W113 group genes into presence in Korean wild aquatic bird (light blue) and in Korean poultry (deep blue). Time points of the separation were inferred from a TMRCA of Korean poultry in each segment. (B) Mean jump counts of HA gene over time were calculated between ecological state and visualized by heat maps.

To examine the genetic diversity of each gene segment, the GMRF Bayesian skyline coalescent analysis was used for all segments of the MS96 group (Korean lineage) and 6 segments of the W113 group (PB2, PB1, PA, NA, M, and NS), which contributed to the generation of the second most prevalent genotype (K4) (Figure 5(A)). Additionally, changes in ecological state were inferred from the HA phylogenetic tree by Markov jump counting (Figure 5(B)). There were three peaks of genetic diversity through all segment in 2002–2003, 2006, and 2009. The first increase peaked in all MS96 genes, followed by the first transition of domestic chicken-to-LBM chicken in 2003. For the second peak, genetic diversity increased in the Korean lineage genes (HA, NP, NA, M, and NS) and Y439 lineage gene (NA). This corresponded to the time of the second transmission from domestic-chicken to LBM-chicken in 2006. The most prominent increase in genetic diversity was third peak involving segments of all W113 group and PB2, PB1, HA, NP, and M genes of the MS96 group in 2009. The time of increase also corresponded to the time that the W113 group was introduced to LBMs and extensive virus exchanges between chickens and ducks in LBMs. Therefore, H9N2 viruses may have evolved through multiple reassortments with viruses originating from wild aquatic birds and frequent viral transmissions between chickens and ducks in LBMs, which might have increased the genetic diversity of the H9N2 viruses.

## Discussion

Implementation of the H9N2 vaccination in commercial chickens in Korea successfully decreased the incidence and severity of disease in commercial poultry [20]. Regardless of the effort, adoption of enhanced biosecurity measures and vaccination were not effectively achieved in backyard production and LBMs. In this study, reconstruction of ecological states on the Bayesian phylogeny demonstrated that the H9N2 virus from commercial chicken farms and viruses originating from wild aquatic birds were disseminated into LBMs and evolved via multiple reassortment events. Additionally, transmission between chickens and ducks in LBMs suggests that the virus had chances to replicate in both species due to the close rearing environment in LBMs. Because LBMs and slaughterhouses are typically the final stages of a retail chain, it was also noted that the transmissions related to slaughterhouses (LBM-chicken to slaughter-chicken and slaughter-chicken to slaughter-duck) indicated that there might be alternative dissemination such as utilizing the same transport with no or inadequate decontamination.

Generally, H9N2 subtype seems to have less reassortment event due to selective constraint and host preference to poultry [33] compared to the wild bird lineage influenza viruses which evolve through a remarkably high rate of reassortment, forming transient genome constellations that rapidly change with no apparent pattern of gene segment association [1,34]. Phylogenetic analysis of Korean H9N2 viruses identified a number of genome constellations (*n* = 29), maintaining HA and NP genes of Korean lineage. To our knowledge, natural reassortment events consistently involving same HA and NP genes of H9N2 AIV has not been previously reported. Our phylogenetic analysis suggests that NP gene of Korean lineage is positively selected. It seems that NP gene picked up on the H9 gene as suggested in a previous study that showed reproducible reassortment involving HA and NP genes originated from a virus lineage during serial passages of human H1N1 and H3N2 [35]. Further studies are required to elucidate the molecular mechanisms leading to the apparent selective advantage of NP gene of Korean lineage over other genetic lineages. It is also noted that the NA gene of Korean H9N2 virus was replaced by only N2 subtype of other lineages, except the A/chicken/Korea/04164/2004(H9N8) virus. Consistent with this finding, Yan et al., showed that the combination of H9 and N2 has better fitness in chickens compared to other NA subtypes including N1 and N3 [36]. It is assumed that H9 and N2 combination has greater viral fitness in poultry than other NA subtypes.

Most of genetic reassortants are likely produced in LBMs. In particular, the phylogenetic analysis determined that three introductions of progenitor gene pools (KJ03, ESD3-3, and W113) contributed to the evolution of the Korean H9N2 viruses. In the case where the AIV originated from Korean ducks, the H3 viruses were transmitted to domestic ducks from wild aquatic birds. Then, the virus primarily circulated in LBM ducks since its identification in 2003 [37]. As experimental infections showed that H3 viruses were shed but caused no clinical signs in chickens [37], LBM chickens could have acted as major hosts, thereby allowing for reassortment with H9N2. In the case with the two AIVs originating from wild aquatic birds, the first identification in poultry was in domestic chickens and ducks with the ESD3-3 and W113 groups, respectively [17,38]. These results also support the argument that the small-scale poultry farms, which are vulnerable to the introduction of viruses originating from wild birds, adopted and transmitted the viruses to LBMs.

Our data indicated that ducks in LBMs adopted viruses originating from wild aquatic birds, and subsequently contributed to the generation and dispersal of the genotype K3, K4, and K5 viruses. Because the Korean H9N2 virus has affected domestic chickens causing clinical signs, chickens have been regarded as the primary host susceptible to the Korean H9N2 viruses. However, frequent detections of the recent genotype (K4) virus in domestic ducks may imply the viruses have acquired sustained transmissibility in domestic ducks due to the reassortment with the genes originating from wild aquatic birds. Likewise, the Chinese BJ94 lineage H9N2 virus had extensive reassortment and adopted internal genes from wild aquatic birds, and became a major genotype (G57) in China [39,40]. It is reported that Muscovy ducks were found to be susceptible to the G57 H9N2 virus in experimental infection [41].

H9N2 viruses have been recognized as donors or recipients of genes with other AIV subtypes, which may increase viral fitness to avian as well as mammalian species by overcoming host barriers [42]. The pathogenicity and transmissibility of newly formed H9N2 genotypes has increased in both chickens and ducks as compared to the initial Korean H9N2 virus [43]. It is also noted that the most recent genotype (K4) generated by reassortment with the W113 group causes high morbidity and has an high intravenous pathogenicity index value (value = 1.15) [44]. In terms of mammalian infection, the initial Korean H9N2 virus was reported to be non-pathogenic to mice [16]. However, some viruses that belong to the genotype K1 as well as newly generated genotypes (K2 and K4) were demonstrated to gain pathogenicity in mice, in particular the genotype K2 showed mortality up to 100% in mice without prior adaptation, possibly due to genetic drift and reassortment [17,45].

Three major increases of genetic diversity in each gene segment of the H9N2 viruses corresponded to transition of ecological state and major genotype. The first and second increases in genetic diversity (2002–2003 and 2006) may have been caused by virus spread from domestic farm chickens to LBM hosts. Because LBMs are where different kinds of domestic poultry and waterfowl congregate and have close contact with each other, frequent virus exchanges between naïve and susceptible hosts could result in increase of genetic diversity. The third increase in genetic diversity in 2009 coincided with emergence of genotype K4 and frequent virus transmission between ducks and chickens in LBM. The growing populations of domestic ducks in small farms that supply the LBMs, and a low level of biosecurity in LBMs, may facilitate the influx of wild bird-origin viruses to LBMs via domestic ducks, rendering the H9N2 virus opportunities for reassortment with a variety of gene pools which could result in increased viral fitness of H9N2 viruses. Our results suggests that LBMs are epicentres for concentrating viruses, and domestic ducks became a major AIV hosts, both of which may contribute to the increased genetic diversity of the H9N2 virus.

In conclusion, our genome sequencing and phylodynamic analysis suggest that the Korean H9N2 viruses spread from commercial chicken farms to the LBM system and evolved through extensive reassortment with viruses originating from domestic ducks and wild aquatic birds. Domestic ducks played a key role in redistributing the progenitor genes that contributed to the evolution of the H9N2 virus. The PB2, PB1, PA, NA, M, and NS genes of the Korean lineage H9N2 virus were replaced with genes originating from wild aquatic birds, and the new genotype virus (K4) became widespread in Korean LBMs. Continuous surveillance and genetic analysis are strongly encouraged in order to investigate how the H9N2 viruses evolve in LBMs. These results also underline a need for implementation of better and comprehensive biosecurity measures at LBMs and small-scale poultry farms to prevent and control of AIV.

## Supplementary Material

Supplemental Material

## Data Availability

Virus sequence data from this study were deposited in Genbank database with the accession numbers MN530096 to MN530927.
