# Supplementary material for: Live bird markets as evolutionary epicentres of H9N2 low pathogenicity avian influenza viruses in Korea
Source: Emerg Microbes Infect. 2020 Mar 17;9(1):616–27. doi: 10.1080/22221751.2020.1738903 (PMC7144223; doi:10.1080/22221751.2020.1738903)
Supplement: Supplemental Material [file TEMI_A_1738903_SM2707.zip › Supplemental tables.docx]

**Supplemental table 1. Time-scaled phylogenetic tree model by viral RNA segments and lineages**

| Segment | Lineage | Model | |
| --- | --- | --- | --- |
|  |  | Substitution and site heterogenicity | Molecular Clock |
| PB2 | Kor | HKY + G | UCLD |
|  | Y439 | GTR + G | UCLD |
|  | EGP1 | GTR + G | UCLD |
| PB1 | Kor | GTR + G | UCLD |
|  | EGP | GTR + G | UCLD |
| PA | Kor | GTR + G | UCLD |
|  | Y439 | GTR + G | UCLD |
|  | EGP1 | HKY + G | UCLD |
|  | EGP2 | GTR + G | UCLD |
| HA | Kor | GTR + G | UCLD |
| NP | Kor | HKY + G | UCLD |
| NA | Kor | GTR + G | UCLD |
|  | Y439 | HKY + G | UCLD |
|  | EGP2 | GTR + G | UCLD |
| M | Kor | GTR + G | UCLD |
|  | Y439 | HKY + G | UCLD |
| NS | Kor | GTR + G | UCLD |
|  | EGP | GTR + G | UCLD |

GTR + G, General time reversible plus gamma distribution; HKY + G, Hasegawa-Kishino-Yano plus gamma distribution; UCLD, Uncorrelated log-normal distribution.

**Supplemental table 2. Time of the most recent common ancestor of H9N2 genes by lineages and segments.**

| Segment | Lineage | Progenitor group | Sub-group | TMRCA |  |  |
| --- | --- | --- | --- | --- | --- | --- |
|  |  |  |  | Mean | 95% HPD interval | |
| PB2 | KOR |  |  | Jan 1995 | Dec 1993 | Dec 1995 |
|  |  | MS96 | clade A | Jan 2000 | Nov 1998 | Dec 2000 |
|  |  | KJ03 | H3 and H6 | Apr 2000 | Dec 1998 | Oct 2001 |
|  |  | MS96 | clade B | Jan 2001 | Nov 1999 | May 2002 |
|  | Y439 | W113 |  | - | - | - |
|  |  |  | Korean wild bird | Jan 2002 | Apr 2001 | Oct 2002 |
|  |  |  | Korean poultry | Jul 2006 | May 2005 | Jul 2007 |
|  |  |  | Korean H9N2 | Jun 2007 | Sep 2006 | Feb 2008 |
|  | EGP1 | ESD3-3 |  | - | - | - |
|  |  |  | Korean wild bird | Nov 2002 | May 2002 | Jun 2003 |
|  |  |  | Korean poultry (H9N2) | Feb 2006 | Oct 2005 | Sep 2006 |
| PB1 | KOR |  |  | May 1995 | Sep 1994 | Mar 1996 |
|  |  | MS96 | clade A | Nov 1999 | Nov 1998 | Jan 2001 |
|  |  | KJ03 | H3 and H6 | Sep 2000 | Dec 1998 | Sep 2002 |
|  |  | MS96 | clade B | Aug 2001 | Mar 2000 | Dec 2002 |
|  | EGP2 | W113 |  | - | - | - |
|  |  |  | Korean wild bird | Sep 2004 | Jan 2004 | Apr 2005 |
|  |  |  | Korean poultry | Dec 2007 | Aug 2007 | May 2008 |
|  |  |  | Korean H9N2 | Apr 2008 | Dec 2007 | Sep 2009 |
| PA | KOR |  |  | Dec 1994 | Sep 1993 | Mar 1996 |
|  |  | MS96 | clade A | Sep 1999 | Mar 1998 | Nov 2000 |
|  |  | KJ03 | H3 and H6 | Aug 2002 | Nov 2000 | Oct 2003 |
|  |  | MS96 | clade B | Feb 2002 | Dec 2000 | Jan 2003 |
|  | Y439 | KJ03 |  | - | - | - |
|  |  |  | Korean poultry | Nov 2002 | Sep 2000 | Oct 2004 |
|  |  |  | Korean H9N2 | Apr 2006 | Apr 2005 | Feb 2007 |
|  | EGP1 | W113 |  | - | - | - |
|  |  |  | Korean poultry | Feb 2004 | May 2003 | Nov 2004 |
|  |  |  | Korean H9N2 | Jan 2005 | Jun 2004 | Aug 2005 |
|  | EGP2 | ESD3-3 |  | - | - | - |
|  |  |  | Korean wild bird | Oct 2002 | Oct 2001 | Jul 2003 |
|  |  |  | Korean poultry | Aug 2007 | Dec 2006 | Mar 2008 |
|  |  |  | Korean H9N2 | Dec 2007 | Jun 2007 | Jun 2008 |
| HA | KOR | MS96 |  | Jun 1996 | Jan 1996 | Oct 1996 |
|  |  |  | clade A | Dec 1999 | Feb 1999 | Sep 2000 |
|  |  |  | clade B | Aug 2003 | Feb 2003 | Dec 2003 |
| NP | KOR |  |  | Sep 1995 | Nov 1994 | May 1996 |
|  |  | MS96 | clade A | No divergence |  |  |
|  |  | KJ03 | H3 and H6 | Nov 2004 | Jul 2003 | Apr 2006 |
|  |  | MS96 | clade B | May 2003 | Jun 2002 | Mar2004 |
| NA | KOR | MS96 |  | Nov 1995 | Nov 1994 | Jun 1996 |
|  |  |  | clade A | Nov 1999 | Spe 1998 | Jan 2001 |
|  |  |  | clade B | Jul 2002 | Apr 2001 | Sep 2003 |
|  | Y439 | KJ03 |  | - | - | - |
|  |  |  | Korean poultry | Jan 1996 | Nov 1992 | Dec 1998 |
|  |  |  | H3 and H6 | Jun 1999 | Oct 1996 | Feb 2002 |
|  |  |  | H9N2 – A | Oct 2006 | Oct 2005 | Aug 2007 |
|  |  |  | H9N2 – B | Nov 2005 | Dec 2004 | Aug 2006 |
|  | EGP2 | W113 |  | - | - | - |
|  |  |  | Korean wild bird | Sep 2004 | Dec 2003 | Jun 2005 |
|  |  |  | Korean poultry | Mar 2008 | Oct 2007 | Aug 2008 |
|  |  |  | Korean H9N2 | May 2008 | Dec 2007 | Sep 2008 |
| M | KOR |  |  | Dec 1994 | Feb 1994 | Oct 1995 |
|  |  | MS96 | clade A | Jun 1999 | Mar 1998 | Sep 2000 |
|  |  | KJ03 | H3 and H6 | Nov 2003 | Apr 2001 | Sep 2005 |
|  |  | MS96 | clade B | Mar 2002 | Jan 2001 | Feb 2003 |
|  | Y439 | W113 |  | - | - | - |
|  |  |  | Korean wild bird | Jan 2000 | Jul 1996 | Aug 2003 |
|  |  |  | Korean poultry | Jun 2007 | Jul 2006 | Feb 2008 |
|  |  |  | Korean H9N2 | Jun 2007 | Aug 2006 | Feb 2008 |
| NS | KOR |  |  | May 1995 | Jun 1994 | Mar 1996 |
|  |  | MS96 | clade A | Oct 1999 | Jun 1998 | Dec 2000 |
|  |  | KJ03 | H3 and H6 | Aug 1999 | Jun 1997 | Jul 2001 |
|  |  | MS96 | clade B | Dec 2001 | Jun 2000 | Mar 2003 |
|  | EGP | W113 |  | - | - | - |
|  |  |  | Korean wild bird | Feb 2001 | Jan 2000 | Feb 2002 |
|  |  |  | Korean poultry | Oct 2007 | Feb 2007 | May 2008 |
|  |  |  | Korean H9N2 | Dec 2007 | May 2007 | Jun 2008 |
